# Bio-oil production from biogenic wastes, the hydrothermal conversion step

**DOI:** 10.12688/openreseurope.14915.1

**Published:** 2022-09-20

**Authors:** Geert Haarlemmer, Anne Roubaud

**Affiliations:** 1 CEA/LITEN/DTCH, Université Grenoble Alpes, Grenoble, 38000, France

**Keywords:** hydrothermal liquefaction, biocrude, biofuel, food wastes, bio-oil, biochar, machine learning

## Abstract

**Background:** Food wastes are an abundant resource that can be effectively valorised by hydrothermal liquefaction to produce bio-fuels. The objective of the European project WASTE2ROAD is to demonstrate the complete value chain from waste collection to engine tests. The principle of hydrothermal liquefaction is well known but there are still many factors that make the science very empirical. Most experiments in the literature are performed on batch reactors. Comparison of results from batch reactors with experiments with continuous reactors are rare in the literature.

**Methods: **Various food wastes were transformed by hydrothermal liquefaction. The resources used and the products from the experiments have been extensively analysed. Two different experimental reactors have been used, a batch reactor and a continuous reactor. This paper presents a dataset of fully documented experiments performed in this project, on food wastes with different compositions, conditions and solvents. The data set is extended with data from the literature. The data was analysed using machine learning analysis and regression techniques.

**Results:** This paper presents experimental results on various food wastes as well as modelling. Aqueous phase recycling allows the re-use of some of the solubilised organics, but this paper shows that after some recycles, the yield is principally in the char yield and not so much in the oil yield. The experimental results were further used to attempt to establish a link between batch and continuous experiments. The molecular weight of bio-oil from continuous experiments appear higher than that of batch experiments. This may be due to the configuration of our reactor.

**Conclusions:** This paper shows how the use of regression models help with understanding the results, and the importance of process variables and resource composition.  A novel data analysis technique gives an insight on the accuracy that can be obtained from these models.

## Plain language summary

Food wastes are an abundant resource that can be valorised by hydrothermal liquefaction. Hydrothermal liquefaction is a process taking place in hot compressed water, typically around 300 °C and above 100 bars. Under these conditions, water is an effective medium to transform organic resources into a black oily product we call biocrude, which can be further upgraded to bio-fuel. The European funded project WASTE2ROAD project aims to demonstrate the full value chain. The principle of hydrothermal liquefaction is well known but there are still many factors that make the science very empirical.

There are essentially two types of reactors in laboratories: generic batch reactors (the majority), and specially designed continuous reactors. Comparison of batch results with experiments with continuous reactors are rare but essential to be able to valorise the large body of data in the literature. Prediction of the results by simulation is often limited as there are many factors that are not measured or cannot be accounted for. This paper presents a dataset of fully documented experiments on food wastes, with different compositions, conditions and solvents. The data from this study is also extended with literature data.

There have been many publications that present correlations or kinetic models for a specific resource. Extrapolation to other resources is hazardous. Recently a different type of modelling, heavily used in Artificial Intelligence and Data Science, makes its way into chemical engineering. These modelling tools are referred to as Machine Learning algorithms. A novel data analysis technique based on machine learning tools give an insight on the accuracy that can be obtained from these models. The experimental results are further used to attempt to establish a link between batch and continuous experiments.

## Introduction

The H2020 European project “Biofuels from WASTE TO ROAD transport” WASTE2ROAD aims to develop a new generation of cost-effective biofuels from a selected range of low cost and abundant biogenic residues and waste fractions. The established consortium covers the full value chain, from waste management, the technological process of transforming waste to advanced biofuels to the assessment of the end-use compatibility of the obtained biofuels. This will be achieved through transformation of a diverse range of waste (and fractions thereof) into intermediate bio-oil, deploying both fast pyrolysis and hydrothermal liquefaction (HTL).

HTL converts biomass compounds in hot compressed water into a biocrude. This biocrude is an oily material containing bio-oil and char. This process has already been known for some time. The developments started in the 80’s in Europe ^1, 2^ and in the United States ^3^. The conversion takes place at temperatures between 300 and 400 °C and at pressures above the saturation pressure to ensure that water remains in the liquid phase, typically above 100 bar. Under these conditions the ionisation of water increases while its polarity decreases, favouring depolymerisation and dehydration of biomass biopolymers to produce hydrophobic compounds ^4^. This process is well adapted for wet resources avoiding an energy consuming step to dry the resource prior to for example combustion, pyrolysis of gasification ^5^. Our previous work on HTL of agro-industrial residues has shown that the biochemical composition of the initial matter is the major parameter influencing conversion efficiency and quality of the product ^6– 8^.

There is a large volume of literature on the basic transformation in batch reactors. Results on continuous reactors show some subtle differences from batch reactors making the results often more difficult to interpret ^6^. The chemistry is complex accentuating the differences between batch end continuous reactors. A significant amount (up to 40 % of the organic dry matter) of the resource is transferred to the aqueous phase product during the transformation. Aqueous phase recycling has an important effect on the results of hydrothermal liquefaction. This has been noted earlier by Déniel *et al*. ^9^ and Biller *et al*. ^10^. From a practical point of view, recycling of the water phase is important to limit the volume of discharged water and to optimise the use of the resource.

The complexity of the resources and large number of process variables make it difficult to fully understand the transformation in one study. The literature is very rich in data that can be exploited to understand the conversion of a particular resource under particular conditions. Multiple studies have been exploited in meta studies where data from a large number of publications is analysed with machine learning tools ^11– 13^. The modelling approach in these papers allows the regression of powerful and accurate models and also allow some basic data analysis. These models are not in equation form and not readily transposable, as they are very complex and relatively meaningless when taken out of context ^14^. The analysis of the data can be pushed further with a game theory approach as was demonstrated by Onsree *et al*. ^15^.

Different types of resources have been considered for HTL conversion in the European funded project WASTE2ROAD. These include food waste, the fermentable fraction of municipal solid waste and its digested counterpart. This paper presents experimental results from these resources under a variety of conditions, and two different reactors. A literature study also identified compatible data that has been used to construct a large homogeneous data set of similar experiments that is analysed with machine learning tools. The objective is to identify plausible explanations for observations.

## Methods

### Resources

The resources used in the project are raw food residues as well as residues from the digestion of food waste. Raw food waste (FW) was sourced from a company restaurant at the Commisariat à l’Energie Atomique et aux Energies Alternatives (CEA) campus in Grenoble. The restaurant which is named ‘H1’ was selected as it is the nearest restaurant (out of three) to our laboratory. The organic fraction of municipal solid waste (FFOM) was supplied by Suez in Montpellier France. This resource is usually used as a feed for methanisation by anaerobic digestion. Energi Gjenvinnings Etatens (EGE) in Norway (Oslo city waste management company) provided anaerobically digested food waste (DFOR) from their methanisation plant.

**Figure 1.**
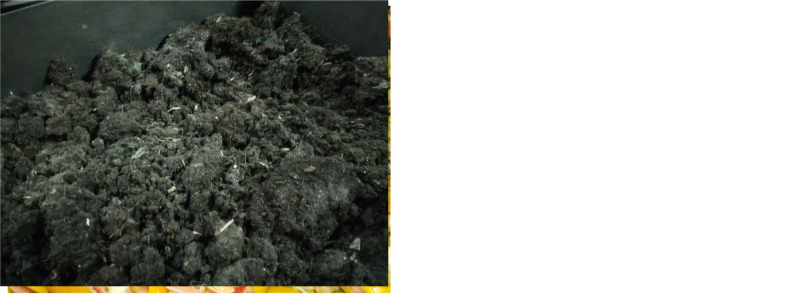
Example of food wastes (FW) from CEA restaurant and Solid bio-residues from biogas reactors (DFOR) provided by EGE, Norway.

The food wastes from CEA are directly taken from the restaurant after the daily service. This waste is a mixture of peels from the food preparation, coffee marc, food that was put on display but not consumed and residues from the plates, Figure 1. The majority of the waste was non-comestible wastes from the food preparation. For economic and legal reasons, as little as possible comestible residues are produced. There is an important daily variability in these wastes. The collection was done during three months period from October to December 2019, in batches of 10 to 20 kg. The collected resources were dried, ground and mixed to ensure they were homogeneous and representative. The DFOR ( Figure 1) and FFOM samples are much more homogeneous and they come from large processing units, for both resources around 50 kg was supplied.

All resources have been analysed by standard methods applicable to foodstuffs, subcontracted to an acredited commercial laboratory Capinov (Landerneau, France). Lipids are quantified by n-hexane extraction ^16^. This method first hydrolyses the resource with hydrochloric acid. Oil is extracted from resulting product by hexane extraction in a Soxhlet device. Fibres are quantified using the methodology as described in the international standards (ISO) ^17, 18^. The sample is first cleaned from lipids by acetone extraction and proteines with digestion with proteinase. The method is based on a series of extractions using a Neutral Detergent, an Acid Detergent a, extraction by sulphuric acid. The resulting values are Neutral Detergent Fibres (NDF), Acid Detergent Fibres (ADF) and Acid Detergent Lignin (ADL). The hemicellulose content is calculated from the difference between NDF and ADF, the cellulose content is the difference between ADF and ADL while ADL is the lignin content.

Proteins are quantified by multiplying Kjeldahl nitrogen by 6.25. The Kjeldahl method only doses ammonial and amine nitrogen (from degraded proteins) but does not quantify nitrates. All organic nitrogen is converted in ammonia that is then quantified by absorption in a boric acid sollution ^19^.

The data include proximate (moisture and ash) and ultimate (elemental) analysis. FFOM and DFOR contain a relatively large amount of ash, some in the form of glass and porcelain particles. Table 1 presents the results of the analyses. The structural compositional analysis was performed by the acredited commercial laboratory Capinov.

**Table 1.** Results of analyses of organic wastes. FFOM, the organic fraction of municipal solid waste; FW, food waste; DFOR, anaerobically digested food waste.

Feedstock description		FFOM	FW1	FW2	DFOR
**STRUCTURAL COMPOSITION**					
**Cellulose**	wt%	30.1	8.6	7.7	8.8
**Hemicellulose**	wt%	5.5	25.3	12.5	9.8
**Lignin**	wt%	7.5	1.9	2.8	24.2
**Proteins**	wt%	9.6	18.2	16.5	29.5
**Lipids**	wt%	3.8	10	11.5	1.3
**Sugars/dry basis (by difference)**	wt%	13.0	27.9	40.1	0
**PROXIMATE ANALYSIS**					
**Total moisture, as received**	wt%	50	90	82	42
**Ash 550 °C**	wt%	22.5	5.1	4.9	28
**ULTIMATE ANALYSIS**					
**Carbon (C)**	wt%	37.7	47.3	43.8	37.6
**Hydrogen (H)**	wt%	5.2	6.3	8.1	5.9
**Nitrogen (N)**	wt%	1.7	3.2	3.2	4.8
**Sulfur (S)**	wt%	0.3	0.1	0.2	0.5
**HEATING VALUE**					
**Higher heating value (HHV)**	MJ/kg	13.8	20.3	20.5	15.9

Chemicals used in this experiment are 2-propanol, acetone, tetrahydrofuran, dichloromethane, ethyl acetate and n-hexane, were acquired from Sigma-Aldrich. Reagent for Karl-Fischer titration, Hydranal Composit 1, was purchased from Honywell. Polycal polystyrene standards for the gel permeation chromatography were purchased from Malvern Panalytics.

### HTL experiments

For each resource, efficiency and product properties were determined by batch experiments. All resources can be treated in batch experiments. The two industrial wastes derived from food waste, FFOM and DFOR, were rich in hard abrasive particles such as glass and ceramics. They cannot be pumped to high pressures without damaging the equipment. For this reason these were only evaluated in batch experiments.

**Figure 2.**
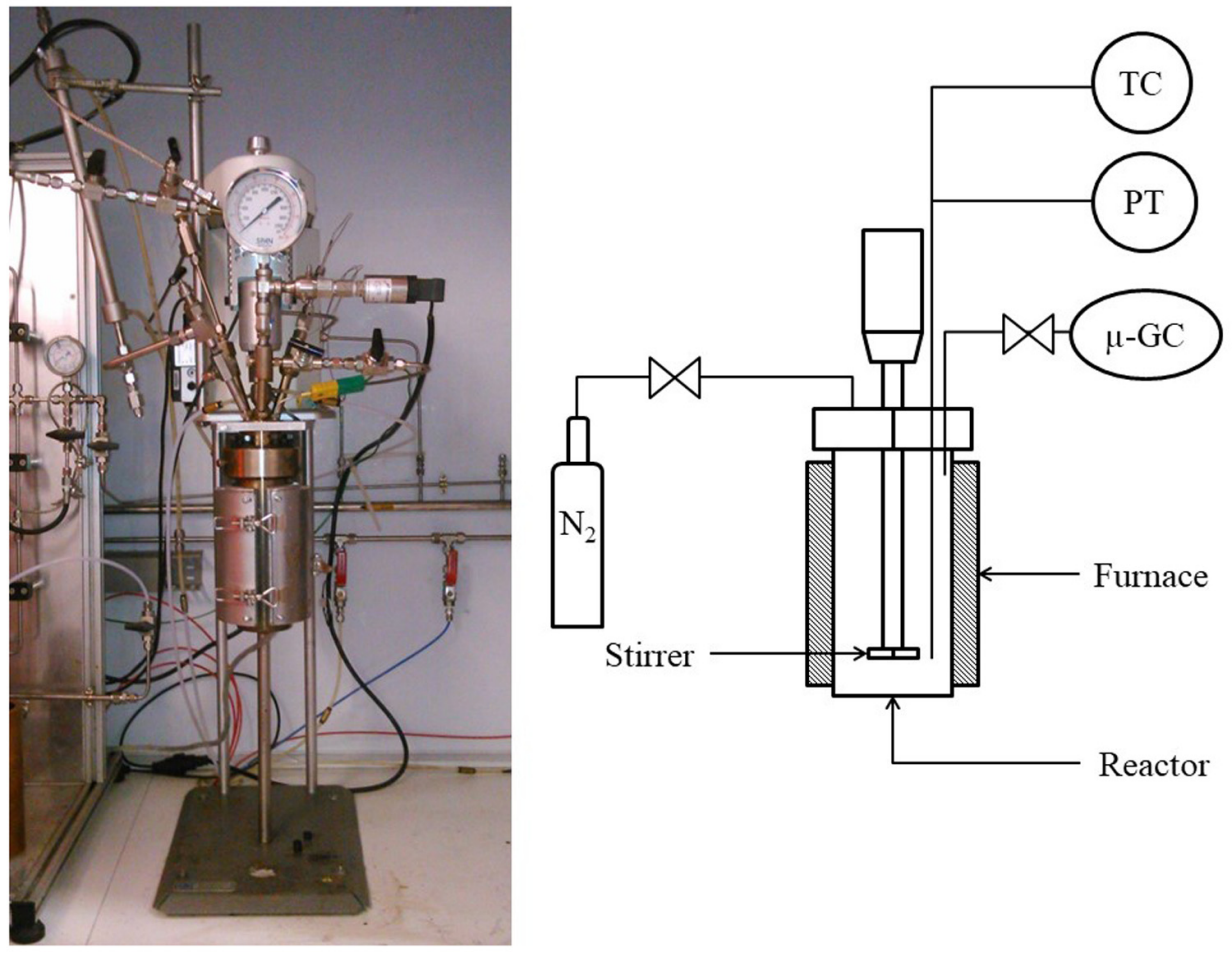
Batch hydrothermal reactor at CEA. TC (Thermocouple), PT (Pressure Transmitter), µ-GC (Connection for the gas analysis by micro chromatography).

Hydrothermal liquefaction experiments were performed in a 0.6 L stainless steel (SS316) stirred batch reactor (Parr Instruments). Figure 2 shows a photo and the schematics of the batch reactor. In a typical experiment, the reactor was filled with 300 g of biomass slurry prepared from 30 g resource and 270 g distilled water. The pH was measured before and after each experiment using a meter. The autoclave was always leak tested, purged of air and pressurized to 10 bar with nitrogen gas. The latter is to ensure a sufficient pressure for gas analysis after the reaction even if not much gas is produced by the reaction. The pressure inside the reactor is a function of the initial amount of nitrogen and the reaction temperature. The amount of water plays a role in that the water occupies space in the reactor that cannot be occupied by the initial and produced gasses. The reactor was heated to the reaction temperature with a constant heating rate of 15 °C/min. Once the reactor had reached the reaction temperature, it was held during a specified time within ± 1 °C of the specified operating temperature. The reactor is stirred at s speed of 600 rpm. After the holding time, the reactor was rapidly cooled to room temperature in 20 min by an air quench.

**Figure 3.**
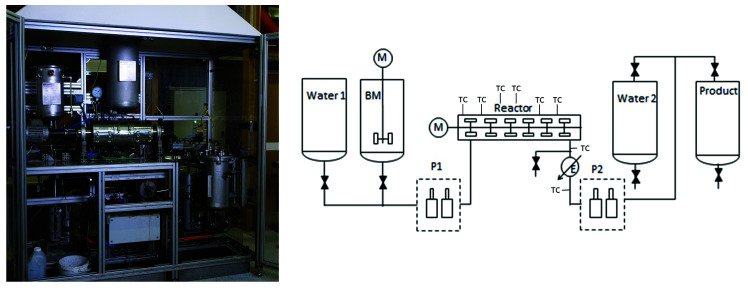
Continuous hydrothermal reactor at CEA. With the biomass tanks (BM), the location of the thermocouples (TC) and the pumps P1 and P2.

Continuous experiments were performed on our 1.5 L/h test bench (TOP Industrie) in similar temperature conditions with selected resource based on the batch experiment results. The temperature setpoints are the same between the two reactors. The batch reactor is heated up together with the reaction mixture while the continuous reactor is continuously heated, fluids are admitted in the already hot reactor. Figure 3 shows a photo and the schematics of the continuous reactor. The installations are described in more detail, including residence time distribution measurements, in Briand *et al*. ^6^ and Briand ^20^. The reactor is heated while operating on a water feed. Once the installation is stable the switch to the biomass slurry tank is made. Operation is typically between 5 and 30 hours. The effective internal volume of the reactor is 0.5 L, leading to an averaged residence time of 20 min. Gas production is measured by the pressure increase on the product tank, followed by gas analysis by micro-chromatography.

### Product recovery batch experiments

**Figure 4.**
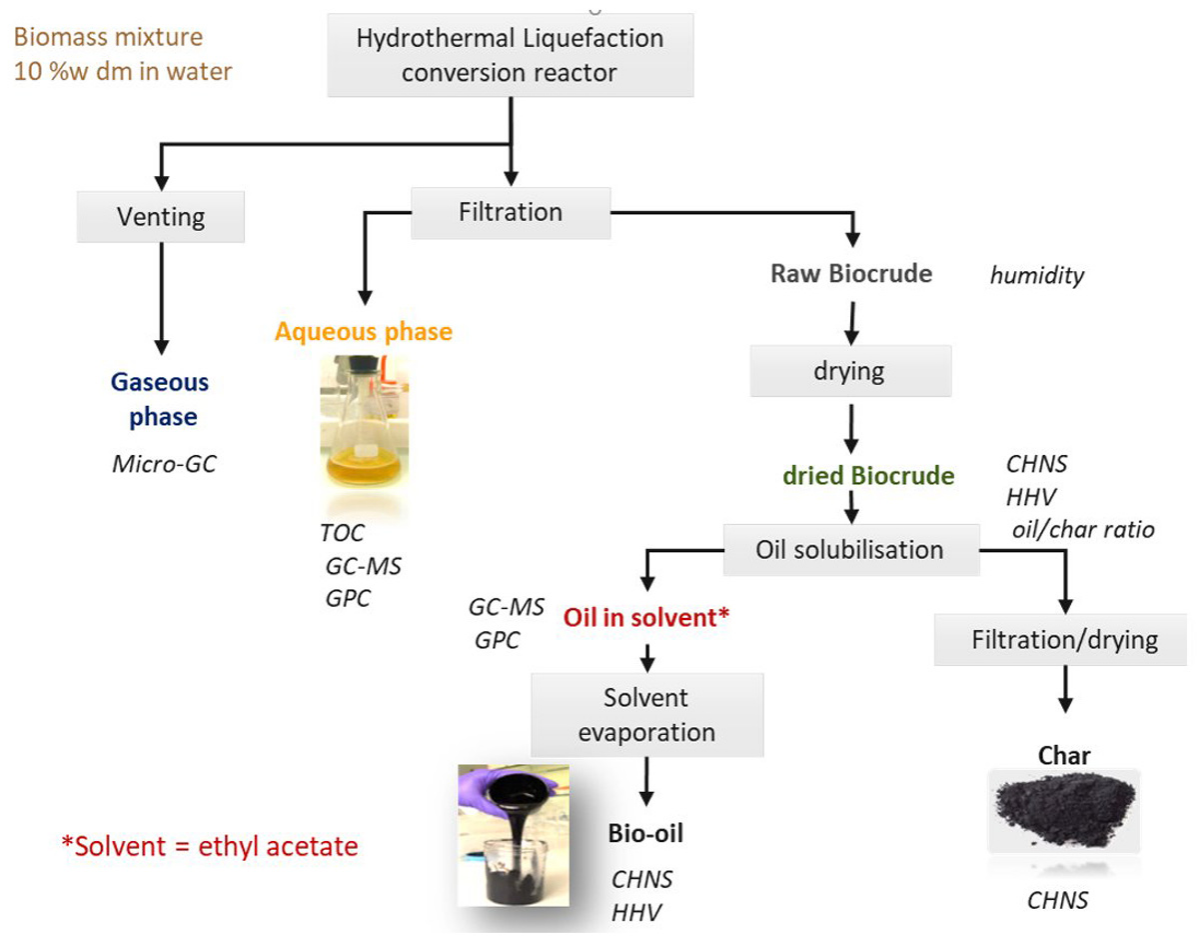
Recovery procedure for products after hydrothermal liquefaction. The specified analysis methods are Carbon-Hydrogen-Nitrogen-Sulphur (CHNS), Higher Heating Value (HHV), Gas Chromatography coupled with Mass Spectroscopy (GC-MS), Gel Permeation Chromatography (GPC) and Total Organic Carbon (TOC).

Before opening, the gas in the reactor is analysed. The reactor was opened and the products were recovered following the procedure given in Figure 4. The content of the reactor was first filtered on a Buchner filter to separate the aqueous phase from the raw organic residue. The raw organic residue is sometimes viscous and sticky. The biocrude was removed from the reactor as best as possible. The biocrude remaining in the reactor is evaluated by comparing the weight of the empty reactor compared to the clean reactor before the experiment. Moisture content of the raw organic residue was estimated depending on the aspect of the product using one of two methods described as follows. Drying at room temperature under air circulation until a stable mass was obtained if efficient for products that are not viscous, with the aspect of a dry powder. Karl Fischer titration using a Schott Instruments Titroline KF was performed when air drying creates a protective crust limiting mass transfer and drying is not possible.

Depending on the proportion of bio-oil and char, the aspect of the raw organic residue can vary from an oily solid to a free-flowing viscous residue. When the char content is high, the bio-oil cannot be directly valorised and solvent extraction is necessary to separate the liquid from the solid fraction. To evaluate the char and oil yields individually, extractions are made on aliquots of the biocrude with different solvents as listed with the experimental data (see *Underlying data*). Two grams (weighed with a precision of 0.1 mg) of biocrude was washed with the respective solvent until the solvent runs off clear. Bio-oil can be recovered after evaporation of the solvent. The char was dried in an oven at 105 °C to remove any residual solvent until a stable weight was obtained (weighed with a precision of 0.1 mg). The proportion of solvent-soluble organics in the biocrude, and therefore the bio-oil yield is the biocrude yield minus its humidity (at the time of the extraction) and char content. This is by no means a substitute to an eventual industrial process, but purely an analytical technique to quantify yields. Biocrudes with high char contents should be considered a product from hydrothermal carbonisation. Biocrudes rich in oil have been “effectively liquefied”.

Mass yields were calculated from the obtained experimental mass of the different phases after the experiments. Yield are defined as the mass ratios between the recovered phases and the dry biomass used in the experiment. In this paper we only report the bio-oil (Y _BO_), char (Y _C_) and gas (Y _G_) yields. The quantity of the organic matter in the water phase is difficult to assess by simple drying as many compounds are volatile. In the literature, the aqueous phase yield is sometimes calculated by difference, closing the mass balance on the organic matter to 100% ^21, 22^. The aqueous phase yield includes the mass balance closure error. Hydration and dehydration reactions make that the overall organic mass balance does not necessarily close to 100%. For this reason we do not report the mass yield of organics in the aqueous phase together with the mass yield of the other phases, since it cannot be accurately determined.

### Product analysis

The gaseous phase was analysed by a micro-chromatograph (Varian Quad CP 4900) that samples the gas from the headspace of the reactor. Permanent gases (O _2_, H _2_, CO, CO _2_ and CH _4_) were analysed by a molecular sieve column using argon as carrier gas. Light hydrocarbons (C _2_H _2_, C _2_H _4_, C _2_H _6_, and C _3_H _8_), and sulphur species (H _2_S and COS) were analysed on a Poraplot-U column using helium as carrier gas.

After the reactor was opened, different recovery procedures described in paragraph 2.3 were applied to analyse the products. The molecular composition of the bio-oil was analysed by a Gas Chromatograph coupled with a Mass Spectrometer, GC-MS (Clarus 500/ Clarus 600S, Perkin Elmer, USA) equipped with a DB-1701 capillary column 60 m × 0.25 mm, 0.25 μm film thickness. A 1 µL sample was injected into the instrument with a split ratio of 10:1. Helium was used as carrier gas. The GC oven temperature was programmed from 45 °C (10 min) to 230 °C at a rate of 6 °C/min, and held at 230 °C during 9.17 min. It was then raised to 250 °C at a rate of 10 °C/min, held at 250 °C during 20 min. The NIST database (NIST/EPA/NIH Mass Spectral Library version 2.0d) was used to identify the peaks.

The recovered biocrude is a viscous material containing bio-oil, char and some water. Air drying is not very effective as the top layer becomes quickly impermeable ^23^. Water content was determined by Karl-Fisher titration based on the reaction between water, sulphur dioxide and iodine on one side and sulphur trioxide and hydrogen iodate on the other (Equipment used Schott Titraline KF).

A portion for analysis is also subjected to azeotropic distillation with toluene to determine the amount of water that can be recovered from the bio-crude. The acidity (Total Acid Number, TAN) was determined by titration according to the method described by Anouti *et al*. ^24^. A total organic carbon analyser (Shimadzu SSM-5000A) quantified the total carbon of the solid and oil samples. A total organic carbon analyser (Shimadzu TOC-L CSH/CSN) quantified total carbon of the aqueous phase.

Gel Permeation Chromatography (GPC), also often referred to as Size Exclusion Chromatography (SEC), was used to characterise the bio-oils in terms of molecular weight. The equipment used is a Viscotek TDA305-010 (Malvern Panalytical). The columns are the T1000, T2500 and T4000 with tetrahydrofuran as eluent. The data is presented in terms of averaged molecular weights in Dalton (Da, equivalent to g/mol). The calibration curve was made from 18 standards with molecular weights ranging from 162 Da to 400 kDa. The chromatogram obtained for the oil is compared to calibration curve to obtain the actual molecular weight distribution. The results are presented averaged by number (Mn) by weight (Mw) and the peak molecular weight (Mp).

### Data collection

The data collected in a single resource is often too small to be of real significance. To complete the dataset we have also included experiments from other authors working with similar food wastes. Even though the literature in HTL is very extensive, zooming in on food wastes, presenting complete data sets severely limits the available data. Data from Motavaf *et al*. ^25^, Bayat *et al*. ^26^, Aierzhati *et al*. ^27^, Evcil *et al*. ^28^ and Yang *et al*. ^29– 31^ are also included. Experiments on soy protein are also included in the data to represent high protein resources ^32– 35^. Table 2 gives on overview of the features, or variables, considered in this study. There are 243 data points in the dataset, small in absolute terms for machine learning and artificial intelligence, but it represents a significant part (if not nearly all) of the available data in the literature. There are more published results on food wastes in the literature, but most of these papers do not present resource composition or fully documented experiments, rendering them unexplainable. It should be noted that Motavaf, Aierzhati and Evcil do not present data for char yield, only bio-oil.

**Table 2.** Range of the data in the included dataset.

Independent variables	Range
Temperature	200-380 °C
Holding time	0-80 min
Dry matter	4–22 %
HHV	13-32 MJ/kg
Ash content	1–30 %
Protein content	2–100 %
Lipids content	1–72 %
Carbohydrates content	1–64 %
Lignin content	2–25 %
Solvents	Ethyl Acetate Dichloromethane Acetone n-Hexane
Method	Order solvent extraction after or before water separation (values 1 and 2 respectively)

**Figure 5.**
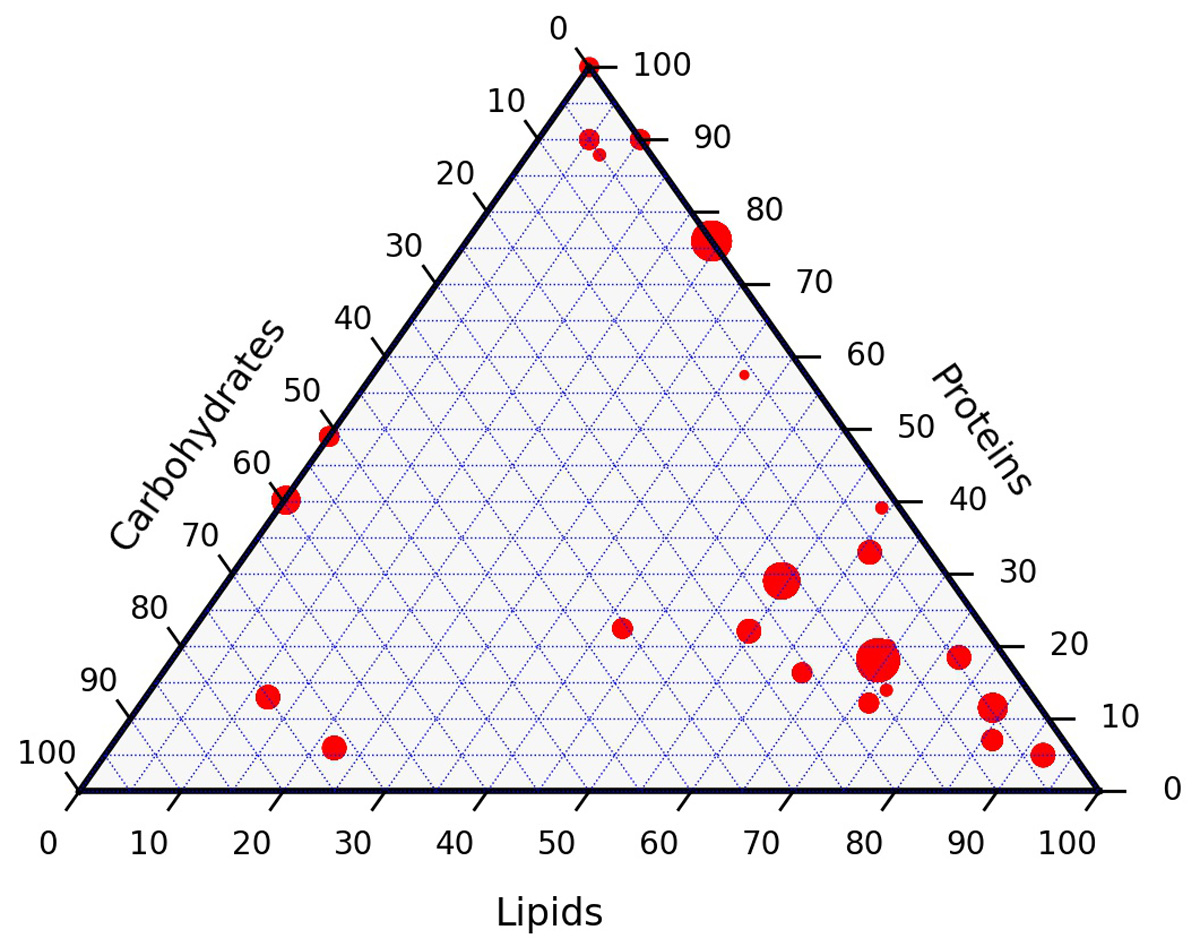
Ternary diagram composition of the resources in this study.

The dry ash free composition of the full data set is presented in Figure 5. Lignin, typically absent or present in low proportions, is added to the carbohydrates. The composition of the resources are used as reported. The dataset covers a wide variety of resources that can be encountered in hydrothermal liquefaction. Individual data points have not been labelled. The size of each point is proportional to the number of data points with this particular composition. The points are not uniformly distributed on the ternary diagram but the resources are fairly representative for typical resources of this category. The full data is available from the *Underlying data* ^36^.

The solvent extraction in our experiments is after separation of the water phase. This is also the case for the data presented by Yang *et al*. ^31^ and Evcil *et al*. ^28^. Other authors introduce the solvent directly into the reactor, this increases the oil yield as some of the organics in the aqueous phase is also extracted and included in the oil yield. A numerical value is attributed to the extraction method, this allows the extraction order to be included in the regression algorithm ^12^. The value of 1 is used when extraction is after water separation, the value of 2 is used when the solvent is added to the reactor after the experiment. Different solvents are characterised by the relative polarity. The values can be found in the *Data availability* section.

### Data analysis with machine learning algorithms

Machine learning is a branch in the large family of artificial intelligence field. The tools in this field are very powerful modelling tools that complement, or even replace, classic polynomial correlations used to model the results from a design of experiment (DOE) approach. The advantage of the machine learning approach is that the data does not need to be highly structured. What counts is the variability of the data and the volume of the dataset. It is also possible to mix pure process parameters (such as the temperature) with indications on the method (as long as we use a numerical value).

Any model created from polynomial correlations, machine learning or any other regression framework devoid of first principles must be taken with caution. Larson’s book on artificial intelligence ^37^ describes the story of the turkey (the original is a chicken from the English philosopher Bertrand Russel) that creates a model by inductive inference of its perfectly comfortable world by observing feeding times and accurately predicting feeding times day after day. One day, on Christmas Eve, this model suddenly no longer works with dramatic consequences. This shows us that any model inferred from observations, how valid they may be, is necessarily limited to the narrow scope of its data validity. The inconvenience of modelling of, what in the end is a chemical reaction without underlying chemical knowledge, is that extrapolation is hazardous. The risk is however somewhat limited when the data set is homogeneous and the model is used for interpolation.

The algorithms used in this study are well known algorithms from the freely available SciKit-Learn library (version 0.24.2) ^38^ and implemented in Python 3.9 (see *Extended data*). Two different regressors have been used in this study, the linear regressor (LinearRegression algorithm) and the random forest regressor (RandomForestRegressor algorithm). The linear regressor produces a very simple linear correlation that is not reputed for its accuracy for arbitrary (and nonlinear) problems but is easy to understand and not prone to overfitting. The random forest regressor is a robust ensemble method based on multiple decision trees. The built in boot-strapping algorithm samples data as it goes along and provides a good protection against overfitting.

Data from studies in any field are subject to uncertainties. In the machine learning and artificial intelligence field, process variables are referred to as features. Uncertainties come from experimental errors due to measurement accuracy and differences in analysis techniques that are not always specified in detail. We may have a very good repeatability of the HTL experiments; it is possible that the resource analyses are not repeatable. Variations also arise from (more or less) subtle differences between resources, as biomass is notoriously variable. Another significant problem arises from different analysis methods. Interpretation becomes much more difficult, when a same property is evaluated with different methods, especially when none of the methods yields an absolute value and are only based on estimates (case of protein content). Less appreciated are uncertainties due to unquantified or unrecognised variables, often referred to as latent features. The accuracy of any model is related to the characteristics of the regression model as well as the data used for the regression. The accuracy that can be obtained from modelling with the data in this study is evaluated using MAPIE (Model Agnostic Prediction Interval Estimator, version 0.3.1) ^39^. This Python library allows the identification of confidence intervals on data modelling with an arbitrary regressor. The theoretical basis of this library is described by Kim *et al*. ^40^ and Barber *et al*. ^41^. The training data contains features ( *X*) and experimental results ( *Y)* with an uncertainty ε, as expressed by Equation 1. The function *µ* is the model function.

Y=μ(X)+εEq. 1

With α being the quartile, each new element has the probability *P* to be in the confidence interval *1-*α, Equation 2.

$$P\left\{Y_{n+1}\in C_{n,\propto }(X_{n+1})\right\}\geq 1-\propto \;Eq. 2$$

The naïve method, as coded in the Gradient Boosting Regressor in the SciKit-Lean Library, creates a model to fit the entire training set for a specified quantile, the fraction of the data being outside the distribution. This technique is prone to overfitting and generates large uncertainty intervals. MAPIE uses the Jacknife+ method, based on a leave-one-out approach. The model is fit successively on the full training set, with one left out. The residual of the left out data point is computed. The regression is then performed on the complete dataset with the confidence interval calculated from the leave-one-out residuals ^41^.

Analysis of the data is also performed using the SHapley Additive exPlanations (SHAP) library, version 0.40.0) ^42^ that supplies algorithms for interpretable AI. The library uses a game theory approach initially proposed by Lloyd Shapely ^43^ and developed by Lundberg *et al*. ^44, 45^ as a Python library. The algorithms in this library allow the evaluation of individual variables and their interactions on the global results as well as individual experiments.

## Results and discussion

Experiments were performed in batch reactors and in a continuous reactor on the same resource at similar conditions. Product yields and oil analyses are presented here. We report in this section the effect of several process parameters such as the temperature, aqueous phase recycling. Experiments in a batch reactor also served to screen more resources and a wider variety of conditions to obtain a larger picture of the transformation. All data underlying the results are available in *Data availability*.

### Continuous and batch experiments

Batch experiments are performed with the feedstocks presented in *Resource* section. Continuous experiments were performed on 98 g/L FW2 enriched with 10 g/L of used cooking oil, named FW2CO. The suspension was stabilised by the addition of 2 g/L xanthane to avoid precipitation in the transfer lines. All experiments were performed at 300 °C and a reaction time of 20 minutes. In the case of the continuous reactor, this means a volume flow rate of 1.5 L/h. Considering the effective volume of the reactor of 0.5 L, this leads to a residence time of 20 minutes. To complete the data, equivalent experiments were experiment was performed in the batch reactor to allow comparison between the two reactors. The results of the batch and the average the continuous experiments are presented in Table 3. The reaction time is assumed to be equivalent to the holding time for the previously presented batch experiments. The reaction time is the averaged residence time in the continuous reactor (volume divided by flow rate). Typical run times in this project were 8 to 20 hours. About 12 kg of biocrude was produced in eight experiments. Precise mass balances have been somewhat difficult to establish. The averages of the experiments are presented in Table 3. The resources FFOM and DFOR produce a lot of char and are not very interesting for HTL. The overall oil yield from FW2CO is somewhat lower for the continuous experiments, but the dispersion between the runs is also higher. The char production is lower in the continuous experiments. The ratio of oil to char is higher for continuous experiments.

**Table 3.** Results from the continuous experiments on FW2. DFOR, digested food wastes; FFOM, The organic fraction of municipal solid waste; FW, food waste.

Experiment	Resource	Conditions	Oil Yield	Char Yield	Oil to Char Ratio	Gas yield
Batch	DFOR	300 °C, 30 min	11.4 ± 0.8 %	47 ± 1 %	0.22 ± 0.2 %	14 ± 1 %
Batch	FFOM	300 °C, 30 min	11 ± 2 %	31 ± 2 %	0.4 ± 0.1 %	13.2 ± 0.1 %
Batch	FW2	300 °C, 30 min	44 ± 2 %	23 ± 2 %	1.9 ± 0.1 %	15 ± 1 %
Batch	FW2CO	300 °C, 20 min	40 ± 4 %	18 ± 0.6 %	2.1 ± 0.2 %	14.6 ± 0.1 %
Continuous	FW2CO	300 °C, 20 min	35 ± 8 %	13.2 ± 3	2.8 ± 0.5	10.3 ± 2

The gas compositions from both batch and continuous experiments on FW2 are presented in Table 4. The gas produced is mainly carbon dioxide with traces of hydrogen and carbon monoxide. There are small differences in the gas quality. It is difficult to estimate a precision of the gas yield in the continuous experiments, as a minor leak is always possible. Reported here is the variability between the experiments without pretending to be an accuracy.

**Table 4.** Gas composition from typical experiments with food waste enriched with used cooking oil FW2CO.

Experiment	Resource	CO _2_	H _2_	CO	CH _4_	C _2_H _x_	C _3_H _x_	H _2_S
		% vol	% vol	% vol	% vol	% vol	% vol	% vol
Batch	FW2CO	89.9	0.8	7.9	0.8	0.22	0.28	0.02
Continuous	FW2CO	85.64	1.17	2.34	0.90	0.06	0.17	0.004

Briand ^20^ has shown that the residence time distribution is relatively flat, the reactor can be simulated by the equivalent of two or three continuously stirred ideal reactors. This means that part of the resources leave the reactor after a short while another faction stays for a longer time. The continuous reactor is at operating temperature and the injected resource is heated quickly. It is possible that high heating rates are more efficient in the depolymerisation of the biomass avoiding the production of primary char by slow pyrolysis of the resource, but no proof of this can be found.

### Bio-oil analysis

**Figure 6.**
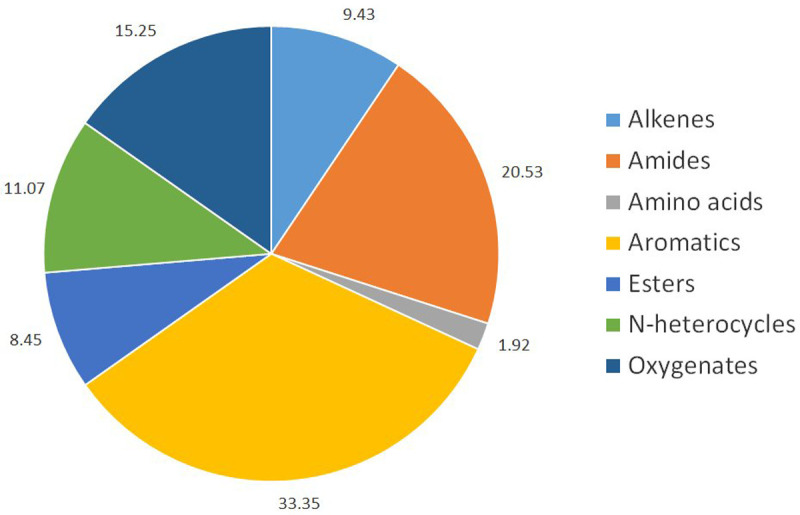
Different chemical families in extracted air-dried bio-oil from the contuous experiments with *food waste enriched with used cooking oil* (FW2CO).

The biocrude was separated in bio-oil and char by solvent extraction with ethyl acetate. Gas chromatography couples with mass spectrometry (GC-MS) analysis was performed for the batch and continuous experiments. The results are presented in terms of areas and are not quantitative. Organic species in the aqueous phase are either oxygenates like alcohol, ketones, cyclic ethers, phenolic species or N species pyrazine and derivatives. The chromatograms and a full list of species are presented in the *Data availability* section. Figure 6 presents the families of molecules that can be identified in the bio-oil from the continuous experiments, with their relative peak areas. More GC-MS results and data can be found in the supplemental material ^36^. The total acid number of this oil is high, 280 ± 25 mg KOH/g oil.

The Gel Permeation Chromatography results in Table 5, show that the bio-oil produced from batch experiments is lighter, of a smaller molecular weight, than those produced from continuous experiments. The observations in Table 3 and section 3.2 that suggested the higher heating rates limited primary char production. Following up on this it appears that the wide residence time distribution in the continuous reactor limits the cracking of the bio-oil compounds and they remain heavier compared to the batch experiments.

**Table 5.** Molecular weights batch and continuous experiments. The results are presented averaged by number (Mn) by weight (Mw) and the peak molecular weight (Mp).

Experiment	Resource	Conditions	Mn (Da)	Mw (Da)	Mp (Da)
Batch	FW2	300 °C, 30 min	382 ± 1	698 ± 36	426 ± 10
Batch	FW2CO	300 °C, 20 min	475 ± 3	745 ± 30	512 ± 4
Continuous	FW2CO	300 °C, 20 min	418 ± 51	1287 ± 800	431 ± 37

### Aqueous phase recycling

**Figure 7.**
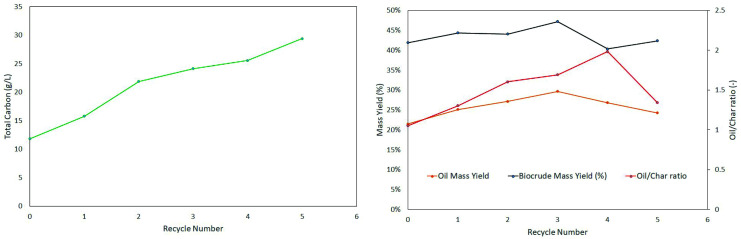
Aqueous recycling experiments.

Hydrothermal liquefaction generates a voluminous aqueous phase waste stream. Recycling this stream into the process helps valorising some of the organics and reducing the volume to be discharged. Recycling of the aqueous phase was done on a series of batch experiments but also in the continuous experiments. The recycle rate is relatively high (>90 %) as the food waste was used in a dried form, losses are limited to small amounts needed for analysis and the losses with the biocrude. In Figure 7 we show how recycling the aqueous phase steadily increases the total carbon content of the aqueous phase waste stream. With successive recycling experiments, the raw biocrude tends to increase its affinity with the aqueous phase and its humidity increases, corrected for in Figure 7 after measurements of the water content with Karl-Fischer titration.

The experiments show that recycling the aqueous phase increases the biocrude yield up to a certain point. At the fourth re-use of the aqueous phase, the oil yield starts to decrease, as does the oil to char ratio, the char production increases steadily. The increased carbon content of the aqueous phase favours char production, apparently by condensation. The averaged molecular weights from the recycling experiments are presented in Table 6. The values of Mn (averaged mole weight by number) and Mp (peak value), are relatively stable. The values of Mw (by weight) goes through a maximum value. This maximum is however nor clearly linked to the observed maximum bio-oil yield in Figure 7.

**Table 6.** Molecular weights recycling experiments with food waste FW2 at 300 °C.

Experiment	Mn (Da)	Mw (Da)	Mp (Da)
0	383	673	419
1	376	**885**	421
2	**352**	781	422
3	374	670	**429**
4	369	666	428
5	360	300	422

### Regression of batch experiments

The resources have been tested in a wide variety of conditions in a batch reactor to evaluate their potential for hydrothermal liquefaction and to better understand their conversion. As there are many experiments with different resources, conditions and solvents, the analysis of the product yields is presented with the aid of machine learning tools. The full data set of the experimental data is presented in the *Underlying data*. The data includes the analysis of the resources, the experimental conditions and the yields.

**Figure 8.**
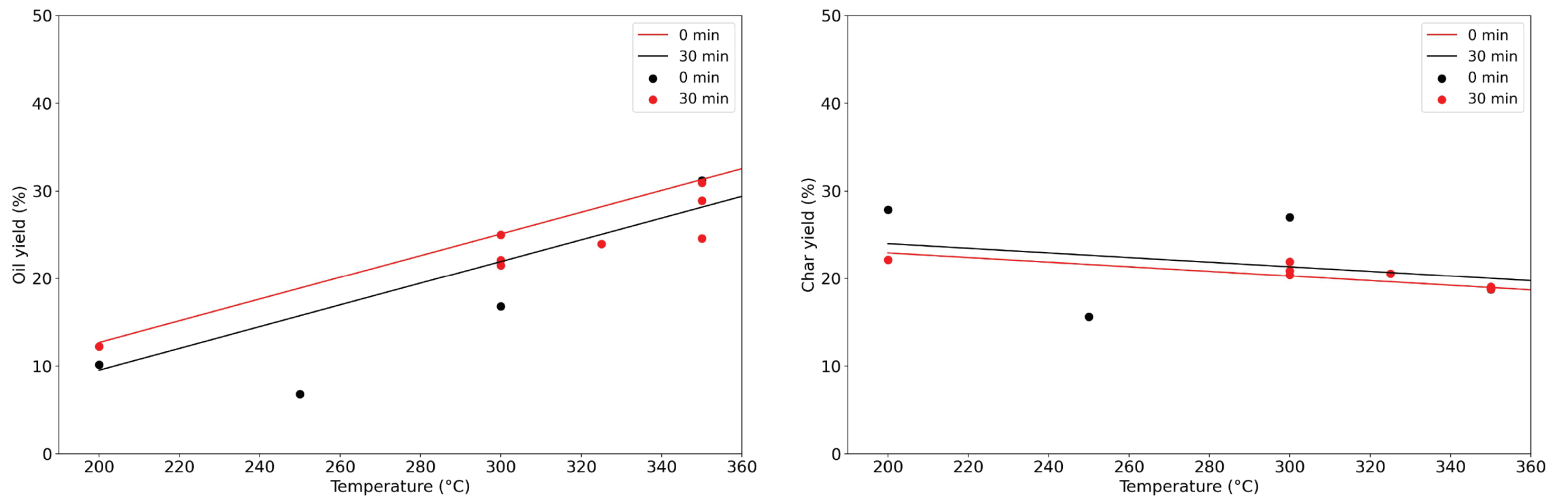
Experimental yields at different temperatures and holding times (oil yield right, char yield left).

Figure 8 presents the results on the food waste labelled FW2 at various temperatures and two different holding times, 0 and 30 minutes. The solid lines represent the results of the linear regression model. In this case, the composition is constant and the only variation are temperature and holding time. As we can see, the linear model does moderately well with the data. The R ^2^ is low, 0.46 for the oil yield and 0.57 for the char yield. Even though the data presents a relatively high dispersion, the general trend is picked up. The equations 3 and 4 describe the linearized behaviour.

Oilyield=−15.3+0.105*HoldingTime+0.12*TemperatureEq. 3

Charyield=29.32−0.035*HoldingTime−0.026*TemperatureEq. 4

**Figure 9.**
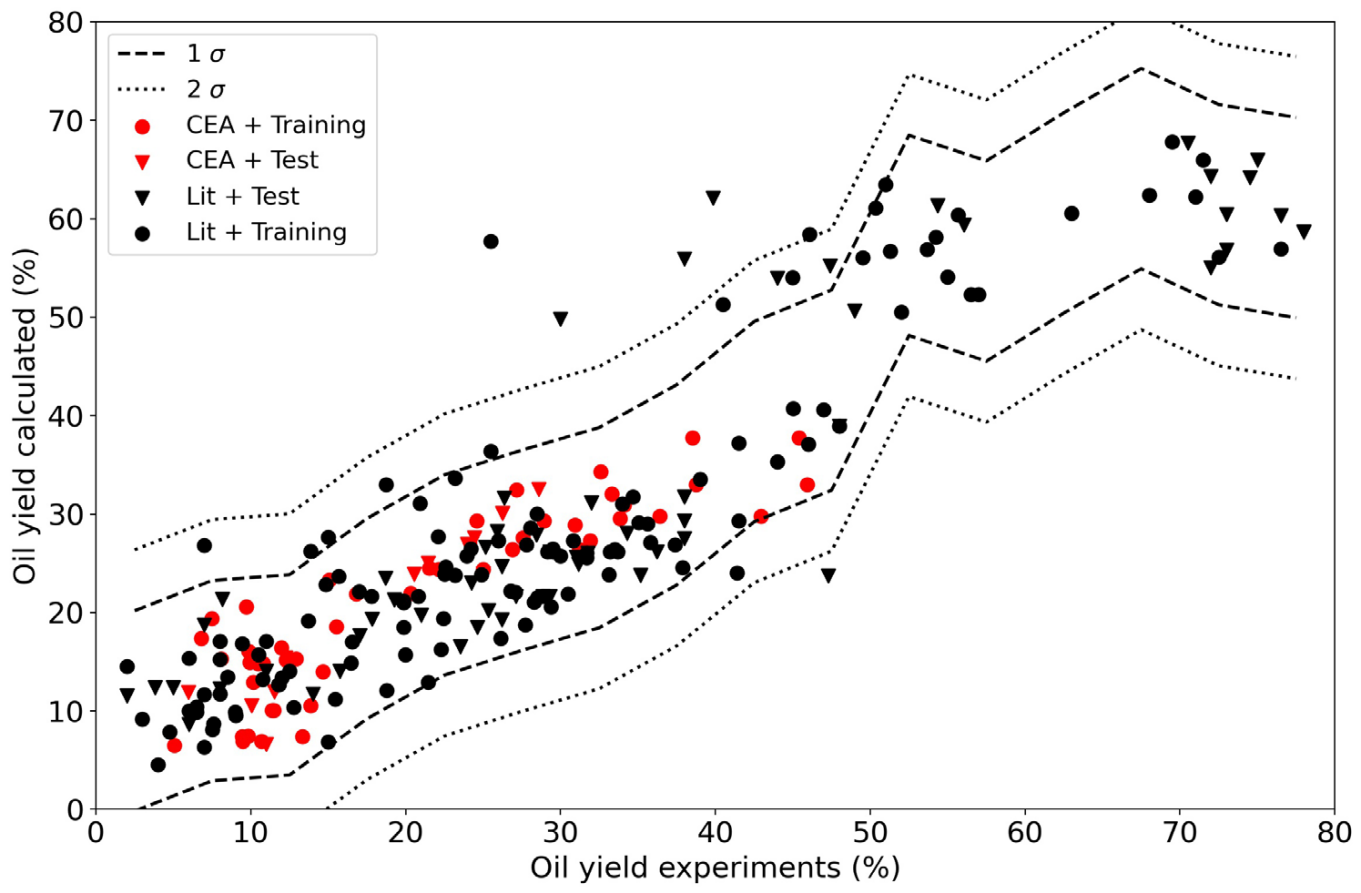
Experimental yields compared to calclated yields using a linear regressor. The data points from the current Waste2Road study are in red (CEA), the data from the literature are in black (Lit). Circles (●) denote training data (80% of the data); triangles (▼) denote test data (20% of the data).

Extending the dataset by including more data from other resources and authors makes the task slightly more complex as compositions and other experimental conditions also play a role. It is no longer possible to plot the results as a function of one particular feature. When we repeat the linear regression for the oil and char yields with the extended data set with all of the data, completed with the literature, we obtain models with relatively low R ^2^ values (84 % for the training data and 73 % for the test data) as shown in Figure 9. This to be expected as a linear model is too simple as has been shown in the past ^7^. A linear model does not do justice to the complexity of the problem.

**Figure 10.**
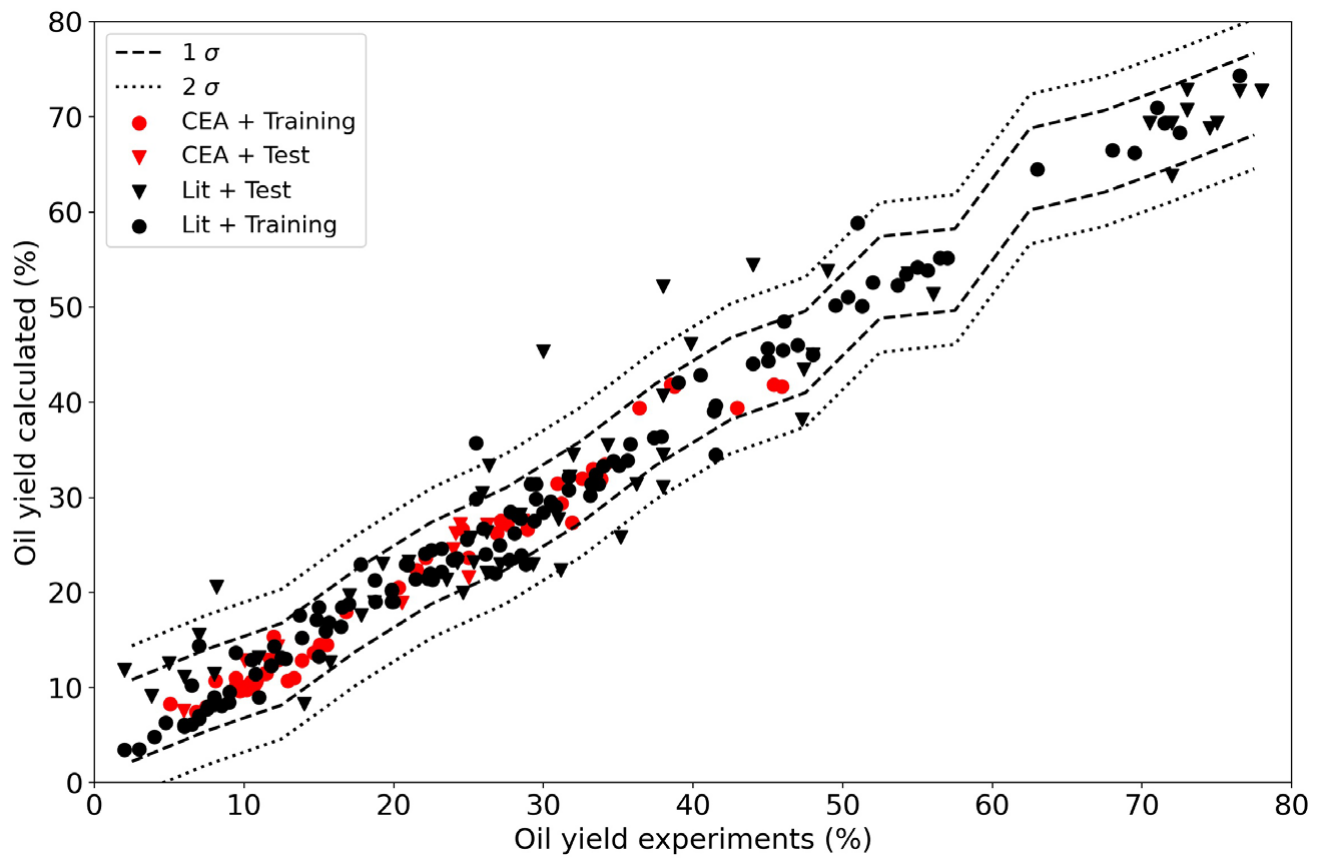
Experimental yields compared to calclated oil yields using a random forest regressor. The data points from the current Waste2Road study are in red (CEA), the data from the literature are in black (Lit). Circles (●) denote training data (80% of the data); triangles (▼) denote test data (20% of the data).

**Figure 11.**
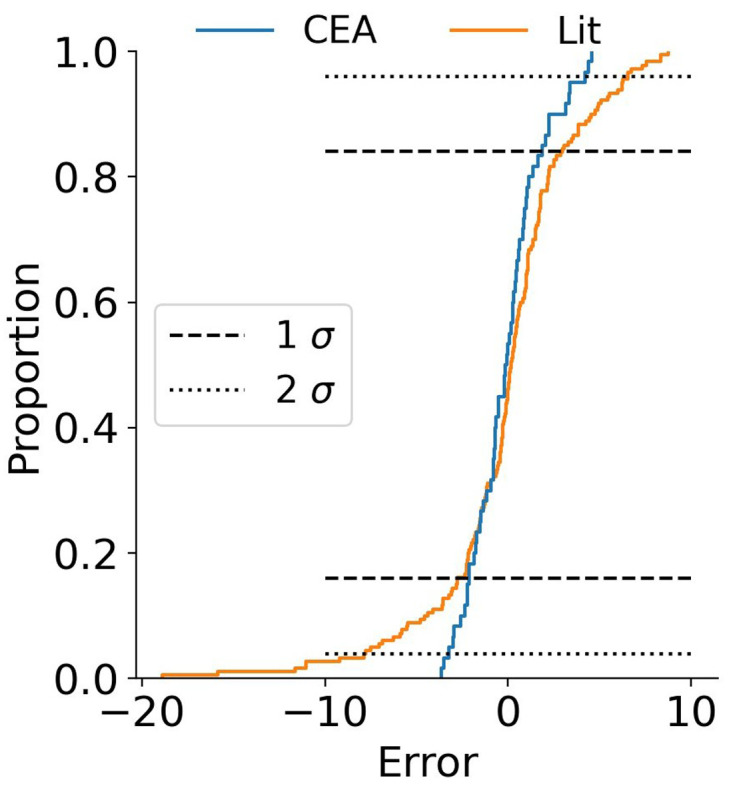
Distribution plot with the random forest regressor of the data produced for this study (CEA) and the literature (Lit).

The results for the random forest regressor are presented in Figure 10. The fit is obviously better with R ^2^ 98 % and 85 % for the training and test data respectively. The confidence interval shrank with the improved fit, 96 % of the data is in a ±5 % interval around the parity. The essence is that the data is not exact, and even though a machine learning algorithm can predict a result, it can only do so with a certain accuracy. The random forest algorithms does a better job than the linear regressor. The distribution of the data is presented in Figure 11. From Table 3 one could conclude that the dispersion in the data in this study is relatively high. This graph shows that the data prepared for this paper has a narrower distribution than the literature data. This could also be inferred visually from Figure 10 but the distribution plot in Figure 11 makes is even clearer.

**Figure 12.**
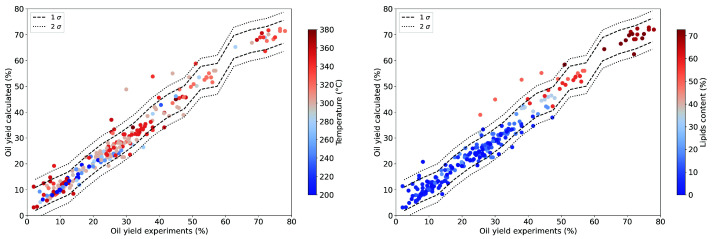
Modelling results (from Python machine learning model) for the oil yield using a random forest regressor with hues for the reactor temperature and lipid content of the resource (Temperature left, Lipid content right).

From these modelling experiments we can conclude that combining datasets makes sense but the interpretation of this type of data should be subject to caution as there are many uncertainties in the characterisation of the experiments, non-quantified inputs as well as measurement error. Figure 12 presents the same data marked with the reaction temperature and the lipid content. The reaction temperature displays no obvious correlation with the oil yield presented in this form. The lipid content is however strongly correlated to the oil yield.

### Feature analysis

**Figure 13.**
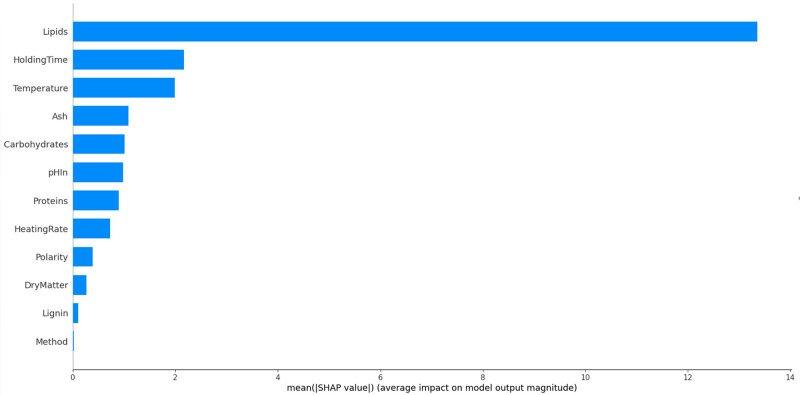
Contributions of the experimental variables on the overall results for bio-oil.

As we have seen, some features like lipid content are strongly correlated with the results; others display a much lower correlation as was shown for the temperature. The influence of each of the variables on the overall result of presented in Figure 13. As is expected, the resource composition, and in particular the lipid content plays a dominant role. Process parameters are less important. These results are valid for the food wastes included in this study, finally on a relatively small sample. Care should be taken to extrapolate these results to other studies and other resources. In a larger study, Li *et al*. ^12^ also found that the role of the lipid content was dominating.

**Figure 14.**
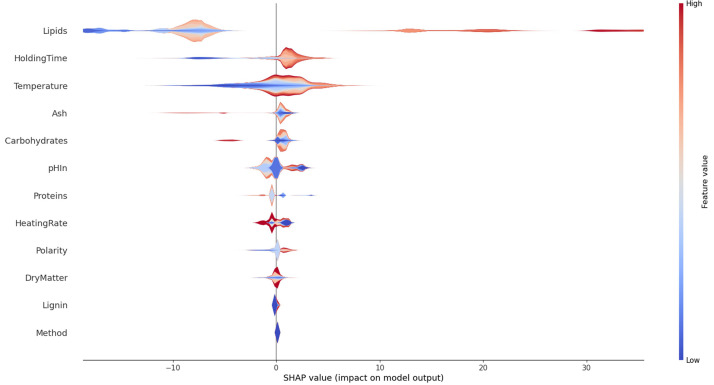
Violin plot of contributions of the experimental variables on the overall results for bio-oil with more details.

The lipid content of the resource is overwhelmingly the most important factor in the HTL conversion in this dataset, before temperature and holding time. The SHAP library offers the possibility to go further in the analysis. The violin pot in Figure 14 shows the importance of features and *how* they influence the result. It can be seen that high lipid contents mostly yield higher than average oil yields. Very low lipid contents yield low oil yields. There is a zone with a moderately negative contribution to the oil yield with higher that averaged lipid yields, showing that there are interactions with other features. The holding time has clearly a positive effect for long times and a negative effect for short times. The analysis method appears to have a very low impact on the result. The ash content is also fairly neutral to the result, except for high values where it has a very negative effect.

**Figure 15.**
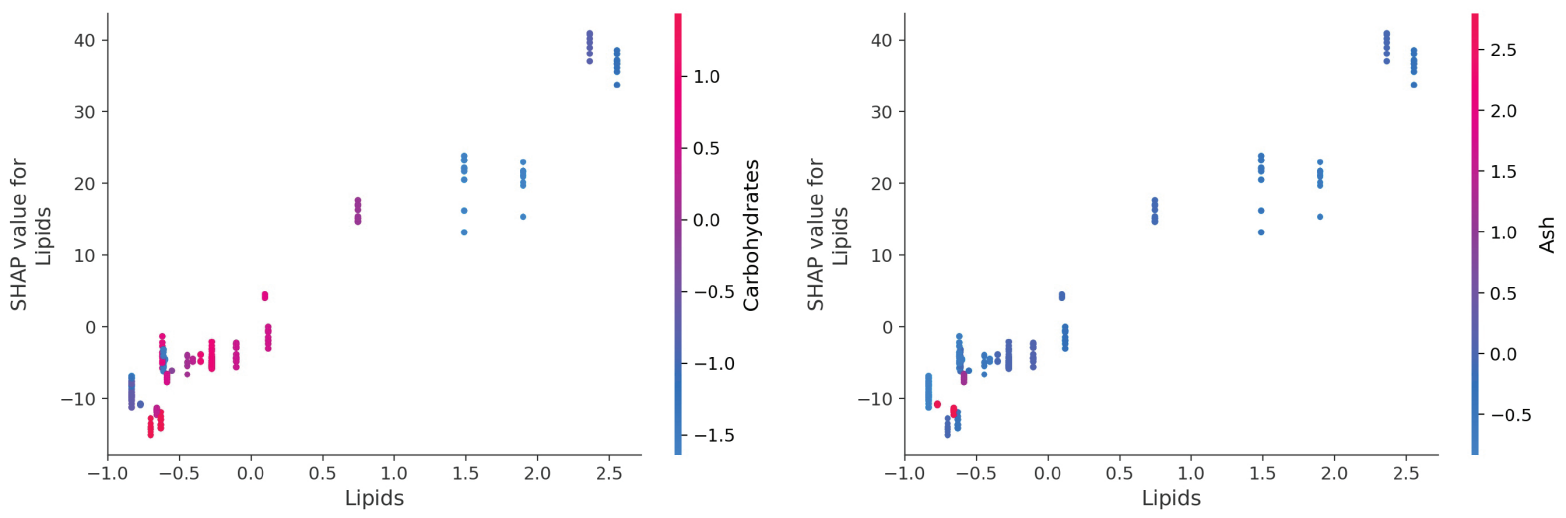
SHAP dependency plots for lipids.

SHAP allows us to go deeper in the analysis. In Figure 14 we have seen how features influence the oil yield. For certain features the image is clear cut as was shown for the holding time. Other features show partial or complete multicolour surfaces, meaning that they do influence the result, but in collaboration with other features. This is especially true for the dry matter content. The lower the feature is ranked in importance the less pronounced the effects are, and subject to statistical noise. The lipid content a faire clear-cut, except for a zone of moderately negative impacts. Figure 15 shows the dependency plot for lipids. Generally, the effect of the lipids is linearly correlated with the oil yield, as can be expected. The points in the graph are coloured with the values for carbohydrates. It can be seen that low values for lipids, also coincide with high values of carbohydrates and ash. The method does not explain why this may be the case. This is most likely due to the fact that high lipid resource and more likely to be low in carbohydrates and ash.

**Figure 16.**
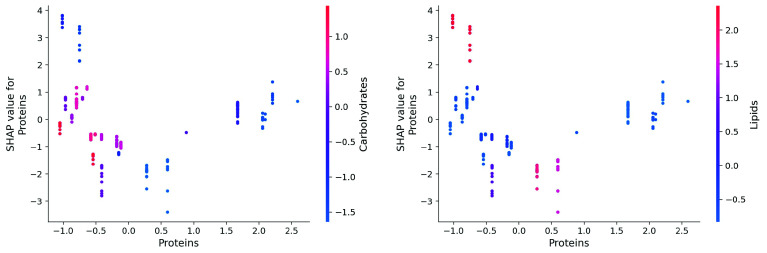
SHAP dependency plots for proteins.

The situation is more complex for proteins that display a different behaviour relative to carbohydrates and lipids as shown in Figure 16. For low protein contents and low carbohydrate content appears to correlate to high lipid content, again, this probably comes from the resources. Very low protein content and high lipids content is good for the oil yield. This is to be expected and compatible with the previous observation. What is more surprising is that moderately low protein content and high lipid content negatively influences oil yields.

## Conclusions

Food wastes are an interesting resource rich in lipids and proteins. At temperatures above 300 °C they produce a sticky to fluid biocrude with an interesting yield of bio-oil, even at low holding times. Lower temperatures favour bio-char regardless of the holding time. The mass yield of the bio-oil obtained is mostly around 40 %. Ash rich resources as digested food wastes (DFOR) or organic fractions of municipal waste (FFOM) from mechanical sorting produce low bio-oil yields and favour char formation.

Continuous experiments have shown that the yields are comparable to batch experiments. The oil to char ratio is an interesting quantity to compare batch and continuous reactor products. The continuous reactor yields an oil to char ratio of nearly three while batch experiments rarely produce a ratio much more than two. The bio-oil from continuous experiments present a higher mass averaged molecular weight (Mw). The higher heating rate may contribute to the higher oil production and oil to char ratio. Mixing in the reactor, leading to a relatively wide residence time distribution, may lead to shorter reaction times for part of the molecules, leading to a higher averaged molecular weight.

Recycling of the process water increase the final carbon concentration in aqueous phase but at the same time increase the biocrude yield (up to 47 % for food wastes for example) so that recycling of the HTL aqueous process water seems a good way to spare water resource and increase the efficiency of the process.

Batch experiments remain a useful tool in the comprehension of hydrothermal liquefaction. Numerous studies in the past have shown that the results from HTL experiments can be described by correlations obtained after carefully designed experimental plans. Data modelling with machine learning tools allow us to establish predictive models with confidence intervals from unstructured data. Experimental data can be enriched with external studies that contribute to the modelling results and increases the accuracy. It is not so easy to transpose actual models from one resource or research to another. They are still essentially used to interpret experimental results.

There are many variables that play a role in hydrothermal liquefaction; these include resource composition, process conditions but also product treatment and analysis. Biomass resources are very complex and an analysis in terms of carbohydrates, proteins, lipids and ash does not do justice to its complexity. It is however an analysis that can be easily performed on all resources. Any single study cannot hope to cover all these parameters in a meaningful way. Combining datasets can be an interesting approach to draw more meaningful conclusions. For this to be possible, authors should take care to fully document their experiments, together with a full resource analysis.

## Ethics and consent

Ethical approval and consent were not required.

## Data Availability

Zenodo: Bio-oil production from biogenic wastes, the hydrothermal conversion step – Data. https://doi.org/10.5281/zenodo.6940211 ^36^. This project includes the following underlying data: HTLYieldData – Publi.xlsx (yield data from the experiments and the literature. Each experiment is labelled to find the corresponding analyser data). Bio-oil production from biogenic wastes, the hydrothermal conversion step - Supp.docx (data from the gas chromatograph with identification of the molecules by mass spectrometry). GCMS.7z (the raw chromatography data created with the program Tubomass 5.4.2 from Perkin Elmer). GPC.7z (raw data from the gel permeatography created with the program Omnisec 5.12.467 from Malvern). µGC Batch.7z (gas analysis of the experiments - Microsoft Excel files). µGC Cont.7z (gas analysis of the continuous experiments - Microsoft Excel files). Data are available under the terms of the Creative Commons Attribution 4.0 International license (CC-BY 4.0). Archived analysis code at time of publication: https://doi.org/10.5281/zenodo.6940211 License: CC-BY 4.0
