# Supplementary material for: Bio-oil production from biogenic wastes, the hydrothermal conversion step
Source: Open Res Eur. 2022 Dec 15;2:111. Originally published 2022 Sep 20. [Version 2] doi: 10.12688/openreseurope.14915.2 (PMC10445818; doi:10.12688/openreseurope.14915.2)
Supplement: Peer review history

# Peer review: Reviewer response for version 2

**DOI:** 10.21956/openreseurope.16647.r30492

**Published:** 2023-01-04

**Authors:** Yuanhui Zhang

**Affiliations:** 1 Department of Mechanical Science Engineering, University of Illinois at Urbana-Champaign, Urbana, IL, USA; 2 Department of Agricultural and Biological Engineering, University of Illinois at Urbana-Champaign, Urbana, IL, USA

The authors have made sufficient revision and rational discussion addressing previous review comments. The manuscript could provide useful information in the area of HTL of food waste. It is recommended to be accepted for indexing.

Is the study design appropriate and does the work have academic merit?

Yes

Is the work clearly and accurately presented and does it cite the current literature?

Partly

If applicable, is the statistical analysis and its interpretation appropriate?

Partly

Are all the source data underlying the results available to ensure full reproducibility?

Partly

Are the conclusions drawn adequately supported by the results?

Partly

Are sufficient details of methods and analysis provided to allow replication by others?

Partly

Reviewer Expertise:

NA

I confirm that I have read this submission and believe that I have an appropriate level of expertise to confirm that it is of an acceptable scientific standard.

# Peer review: Reviewer response for version 2

**DOI:** 10.21956/openreseurope.16647.r30493

**Published:** 2023-01-04

**Authors:** Daniele Castello

**Affiliations:** 1 AAU Energy, Aalborg University, Aalborg, Denmark

I have read the authors's replies to my previous reviews and found all answers adequate.

 I would therefore advise the indexing of the manuscript in its revised form.

Is the study design appropriate and does the work have academic merit?

Yes

Is the work clearly and accurately presented and does it cite the current literature?

Yes

If applicable, is the statistical analysis and its interpretation appropriate?

Yes

Are all the source data underlying the results available to ensure full reproducibility?

Partly

Are the conclusions drawn adequately supported by the results?

Yes

Are sufficient details of methods and analysis provided to allow replication by others?

Partly

Reviewer Expertise:

Biomass, hydrothermal liquefaction, hydroprocessing, upgrading to fuels, drop-in fuels

I confirm that I have read this submission and believe that I have an appropriate level of expertise to confirm that it is of an acceptable scientific standard.

# Peer review: Reviewer response for version 1

**DOI:** 10.21956/openreseurope.16118.r30148

**Published:** 2022-10-20

**Authors:** Yuanhui Zhang

**Affiliations:** 1 Department of Mechanical Science Engineering, University of Illinois at Urbana-Champaign, Urbana, IL, USA; 2 Department of Agricultural and Biological Engineering, University of Illinois at Urbana-Champaign, Urbana, IL, USA

This manuscript reports an experimental and analytical/modeling study of bio-oil production from food waste via hydrothermal liquefaction (HTL). The experiment was well designed and new experimental data were obtained. An innovative part of the paper is using SHAP (a software) analysis to model the HTL bio-oil production affected by operating conditions (reaction temperature and holding time) and biochemical compositions of food waste. The SHAP model includes 12 variables which is very comprehensive and intriguing. The results show good agreement of the experiment data and the SHAP analysis. Given the complexity of the chemical reactions in HTL, most of HTL studies did cover such a comprehensive range of variable. To make the SHAP analysis more convincing, it is suggested by this reviewer to include more data, especially those of related to the variables affecting the HTL performance.    

 The SHAP database could be further expanded to other feedstocks similar to food waste such as manure and algae, with similar biochemical composition (category-wise) for HTL. There are a wealth of lab-scale HTL experimental studies, and a few pilot-scale HTL studies in the past five years, which were largely missed in the current version of the manuscript. Works of a few groups with sustained HTL research could help to strengthen the SHAP analysis – those groups including my own lab at University of Illinois at Urbana-Champaign, Savage’s lab in Penn State University, and so on. I listed some examples of publications related to the HTL work from my own lab (for my own simplicity), to show there are a broad range of data and work related to evaluate the HTL performance, which by no means is required or exclusive.

 Listed below are some example references for HTL data collection based on my own work, but the authors should engage more broadly with the literature.

Is the study design appropriate and does the work have academic merit?

Yes

Is the work clearly and accurately presented and does it cite the current literature?

Partly

If applicable, is the statistical analysis and its interpretation appropriate?

Partly

Are all the source data underlying the results available to ensure full reproducibility?

Partly

Are the conclusions drawn adequately supported by the results?

Partly

Are sufficient details of methods and analysis provided to allow replication by others?

Partly

Reviewer Expertise:

hydrothermal liquefaction of wet waste.  waste-to-energy.

I confirm that I have read this submission and believe that I have an appropriate level of expertise to confirm that it is of an acceptable scientific standard, however I have significant reservations, as outlined above.

## References

[1] : Effect of ash on hydrothermal liquefaction of high-ash content algal biomass. *Algal Research* .2017;25: 10.1016/j.algal.2017.05.010 297-306 10.1016/j.algal.2017.05.010

[2] : Physical pretreatments of wastewater algae to reduce ash content and improve thermal decomposition characteristics. *Bioresour Technol* .2014;169: 10.1016/j.biortech.2014.07.076 816-820 10.1016/j.biortech.2014.07.076 25123982

[3] : Biocrude Oil Production through the Maillard Reaction between Leucine and Glucose during Hydrothermal Liquefaction. *Energy & Fuels* .2019;33(9) : 10.1021/acs.energyfuels.9b01875 8758-8765 10.1021/acs.energyfuels.9b01875

[4] : Nitrogen Migration and Transformation during Hydrothermal Liquefaction of Livestock Manures. *ACS Sustainable Chemistry & Engineering* .2018;6(10) : 10.1021/acssuschemeng.8b03810 13570-13578 10.1021/acssuschemeng.8b03810

[5] : Extract Nitrogen-Containing Compounds in Biocrude Oil Converted from Wet Biowaste via Hydrothermal Liquefaction. *ACS Sustainable Chemistry & Engineering* .2016;4(4) : 10.1021/acssuschemeng.5b01645 2182-2190 10.1021/acssuschemeng.5b01645

[6] : Hydrothermal liquefaction of protein-containing feedstocks.2018; 10.1016/B978-0-08-101029-7.00004-7 127-168 10.1016/B978-0-08-101029-7.00004-7

[7] : Effects of the extraction solvents in hydrothermal liquefaction processes: Biocrude oil quality and energy conversion efficiency. *Energy* .2019;167: 10.1016/j.energy.2018.11.003 189-197 10.1016/j.energy.2018.11.003

[8] : Hydrothermal liquefaction of typical livestock manures in China: Biocrude oil production and migration of heavy metals. *Journal of Analytical and Applied Pyrolysis* .2018;135: 10.1016/j.jaap.2018.09.010 133-140 10.1016/j.jaap.2018.09.010

[9] : Development of a mobile, pilot scale hydrothermal liquefaction reactor: Food waste conversion product analysis and techno-economic assessment. *Energy Conversion and Management: X* .2021;10: 10.1016/j.ecmx.2021.100076 10.1016/j.ecmx.2021.100076

[10] : Establishment and performance of a plug-flow continuous hydrothermal reactor for biocrude oil production. *Fuel* .2020;280: 10.1016/j.fuel.2020.118605 10.1016/j.fuel.2020.118605

[11] : Recovery of reducing sugars and volatile fatty acids from cornstalk at different hydrothermal treatment severity. *Bioresour Technol* .2016;199: 10.1016/j.biortech.2015.08.043 220-227 10.1016/j.biortech.2015.08.043 26316401

[12] : Effects of reaction temperature and reaction time on the hydrothermal liquefaction of demineralized wastewater algal biomass. *Bioresource Technology Reports* .2021;14: 10.1016/j.biteb.2021.100679 10.1016/j.biteb.2021.100679

# Response

**Published:** 2022-11-28

**Authors:** Geert Haarlemmer

**Affiliations:** Atomic Energy and Alternative Energies Commission, France, France

*Dear Reviewer,*

*Thank you for taking the time to read and review our paper. We have made significant improvements to the paper. Please find responses to your remarks below.*

 This manuscript reports an experimental and analytical/modeling study of bio-oil production from food waste via hydrothermal liquefaction (HTL). The experiment was well designed and new experimental data were obtained. An innovative part of the paper is using SHAP (a software) analysis to model the HTL bio-oil production affected by operating conditions (reaction temperature and holding time) and biochemical compositions of food waste. The SHAP model includes 12 variables which is very comprehensive and intriguing. The results show good agreement of the experiment data and the SHAP analysis. Given the complexity of the chemical reactions in HTL, most of HTL studies did cover such a comprehensive range of variable. To make the SHAP analysis more convincing, it is suggested by this reviewer to include more data, especially those of related to the variables affecting the HTL performance.    

*Response: Variables affecting the HTL performance are mostly included in this study, the main being composition, temperature and holding time. Other less important parameters such as heating rate and dilution are also included. We were surprised to see that finally the solvent and extraction order have a limited effect on the results.*

 The SHAP database could be further expanded to other feedstocks similar to food waste such as manure and algae, with similar biochemical composition (category-wise) for HTL. There are a wealth of lab-scale HTL experimental studies, and a few pilot-scale HTL studies in the past five years, which were largely missed in the current version of the manuscript. Works of a few groups with sustained HTL research could help to strengthen the SHAP analysis – those groups including my own lab at University of Illinois at Urbana-Champaign, Savage’s lab in Penn State University, and so on. I listed some examples of publications related to the HTL work from my own lab (for my own simplicity), to show there are a broad range of data and work related to evaluate the HTL performance, which by no means is required or exclusive.

*Response: Yes, point taken, the more data the better. In this paper we want to limit ourselves to a particular type of feedstock that does not receive much attention. There are many HTL papers on algae. Microalgae are however quite different from food wastes in terms of composition and physical aspect. We prefer to stick to food wastes and they are a relevant waste that does not receive much attention in the literature.*

 Listed below are some example references for HTL data collection based on my own work, but the authors should engage more broadly with the literature.

*Response: Thank you for the references. There are some that I did not know.*

# Peer review: Reviewer response for version 1

**DOI:** 10.21956/openreseurope.16118.r30147

**Published:** 2022-10-17

**Authors:** Daniele Castello

**Affiliations:** 1 AAU Energy, Aalborg University, Aalborg, Denmark

The present manuscript reports a comprehensive analysis of HTL biocrude production from a number of organic waste, including fresh food waste, organic fraction of municipal solid waste and fermented digestate.

 The manuscript is in general well-written and presents a number of interesting results. However, there are some parts that need to be presented in a much more concise fashion, while some others are excessively condensed and lack the necessary space to be fully developed. The manuscript is indeed trying to summarize several topics, i.e. the differences between batch and continuous processes, the effect of aqueous phase recirculation and finally a machine learning model for HTL built from a large dataset. Probably it is too much information for a single manuscript.

1. An important issue is the study on aqueous phase recirculation. In my opinion, this is a not well-placed research question. Indeed, the kind of organic waste taken into account are by their nature wet (potentially even up to 70-80%). In these conditions, aqueous phase recirculation cannot be established, because it would bring to accumulate water in the system. On the other hand, a crucial advantage of HTL over other thermochemical technologies is that the feedstock does not need pre-drying. The authors could explore aqueous phase recirculation because their feedstock was dried beforehand, but this does not correspond to what anyone would do in practice. The authors therefore need to better explain the motivation behind aqueous phase recirculation and how it could be realistically established in a real plant.
2. Regarding the experimental procedure, there are some weak points. How could the biocrude remaining in the reactor be evaluated just by weight difference? Is it assumed that there are no residual solids in the reactor? This operation is normally achieved by washing the internal part of the reactor with a solvent and then separating and quantifying the products.
3. How was the amount of produced gas quantified?
4. What was the original moisture content of the feed, before pre-drying?
5. “Biocrude with high char contents could be considered a product from hydrothermal carbonization” (p. 6). This definition is quite weak and I suggest removing or rephrasing, as there are no fixed boundaries between different processes and, especially, the utilization of the products depends on the solutions utilized on the production plant and not merely by their amounts. For example, scaling up the process to a continuous flow unit could feature some form of on-line filtration allowing to recover important amounts of biocrude.
6. The section “Product analyses” tends to be redundant with respect to the section “Product recovery batch experiments”. For example, Karl-Fischer titration is described again. Moreover, azeotropic distillation is not defined. There seem to be two almost similar TOC apparatuses: please have it checked.
7. “A numerical value is attributed to the extraction method (…)”: this is not very clear, especially it is not clear how this value affects the regression algorithm.
8. The section “Data analysis with machine learning algorithms” seems quite didactical, even with some funny anecdote, like the story of the turkey. I would advise more conciseness, reporting only information necessary to an average-skilled reader to understand and reproduce the methodology.
9. In Fig. 5 caption, it should be specified that carbohydrates include lignin.
10. The first part of the section “Continuous and batch experiments” belongs to Materials and Methods and not to Results.
11. Units in Table 3 are wrong. For example, oil to char ratio is not a percentage, while char and gas yields in the last row are. The unit for yields should be made explicit (wt. %). A carbon balance to verify closure is highly advised.
12. One of the differences between batch and continuous is the long-lasting thermal transient in batch operations, which is not present in continuous.
13. Fig. 6 is clearly wrong, as it is identical to Fig. 4. It should report the GC-MS analysis, instead.
14. The caption in Fig. 7 is insufficient. Please explain what the left (a) and right (b) plots represent.
15. Eq. 3 and 4 look misleading. There should be a precise indication of the validity limit, especially for residence time. Moreover, it is awkward that a dependence is stated for residence time, when only two values of this variable were tested.
16. At page 13, the paragraph: “From table 3 one could conclude (…) makes is even clear” is not clear and needs to be rephrased.
17. The legend in Fig. 8 is unclear. Its caption should report (a) and (b) instead of “left” and “right” (same also in Fig. 12).
18. Fig. 9 and 10 could be improved by drawing the parity line. In Fig.9 caption correct “calclated". Somewhere in the manuscript, a full account of the sources utilized to produce the dataset should be given.
19. The plot in Fig. 11 is not clear. What do the axes mean? What are their units of measure?
20. What is “SHAP”?
21. The discussion in section “Feature analysis” is too much condensed and would probably need more space to be properly expanded. I would suggest reducing the number of graphs and selecting the information to comment.
22. The reported explanation on why low values of lipids coincide with high values of carbohydrates is nicely obvious: “low values for lipids coincide with high values of carbohydrates due to the fact that high values of lipids correspond to low values of carbohydrates”. A more convincing explanation should be found, if any.
23. Fig. 15 and 16 have insufficient captions, as each subplot must be identified and described. Moreover, the units of the represented variables must be given.
24. In the conclusions, “recycling of the HTL aqueous process water seems a good way to spare water resource”. This is misleading, as in a real process you would receive wet biomass, so no external water should be needed. On the contrary, there is a problem with the disposal of process wastewater according to environmental standards.

Is the study design appropriate and does the work have academic merit?

Yes

Is the work clearly and accurately presented and does it cite the current literature?

Yes

If applicable, is the statistical analysis and its interpretation appropriate?

Yes

Are all the source data underlying the results available to ensure full reproducibility?

Partly

Are the conclusions drawn adequately supported by the results?

Yes

Are sufficient details of methods and analysis provided to allow replication by others?

Partly

Reviewer Expertise:

Biomass, hydrothermal liquefaction, hydroprocessing, upgrading to fuels, drop-in fuels

I confirm that I have read this submission and believe that I have an appropriate level of expertise to confirm that it is of an acceptable scientific standard, however I have significant reservations, as outlined above.

# Response

**Published:** 2022-11-28

**Authors:** Geert Haarlemmer

**Affiliations:** Atomic Energy and Alternative Energies Commission, France, France

*Dear Reviewer,*

*Thank you for taking the time to read and review our paper. We have made significant improvements to the paper. Please find responses to your remarks below.*

 The present manuscript reports a comprehensive analysis of HTL biocrude production from a number of organic waste, including fresh food waste, organic fraction of municipal solid waste and fermented digestate. The manuscript is in general well-written and presents a number of interesting results. However, there are some parts that need to be presented in a much more concise fashion, while some others are excessively condensed and lack the necessary space to be fully developed. The manuscript is indeed trying to summarize several topics, i.e. the differences between batch and continuous processes, the effect of aqueous phase recirculation and finally a machine learning model for HTL built from a large dataset. Probably it is too much information for a single manuscript.

*Response: Thank you for your review. You highlighted quite a few points that needed addressing. We feel that the manuscript has been greatly improved. You find a point by point response below.*

 1.    An important issue is the study on aqueous phase recirculation. In my opinion, this is a not well-placed research question. Indeed, the kind of organic waste taken into account are by their nature wet (potentially even up to 70-80%). In these conditions, aqueous phase recirculation cannot be established, because it would bring to accumulate water in the system. On the other hand, a crucial advantage of HTL over other thermochemical technologies is that the feedstock does not need pre-drying. The authors could explore aqueous phase recirculation because their feedstock was dried beforehand, but this does not correspond to what anyone would do in practice. The authors therefore need to better explain the motivation behind aqueous phase recirculation and how it could be realistically established in a real plant.

*Response: You are right of course. We are in an academic context where the resources have been dried to allow preconditioning of the resources. The initial idea was to shed some light on the chemistry but after review we have decided to remove this section and it is not really important. The data will remain available.*

 2.    Regarding the experimental procedure, there are some weak points. How could the biocrude remaining in the reactor be evaluated just by weight difference? Is it assumed that there are no residual solids in the reactor? This operation is normally achieved by washing the internal part of the reactor with a solvent and then separating and quantifying the products.

*Response: This only concerns the batch reactor. The text was amended to explain the reactor was dried in an oven at around 50 °C before weighing. A relatively small amount of biocrude remains, typically less than one gram. This amount is taken into account in the mass balances.*

 3.    How was the amount of produced gas quantified?

*Response: The gas production was calculated by the ideal gas law, from the pressure increase during the experiment as well as the dissolved carbon dioxide as calculated by Henry’s law. These equations have been included in the text.*

 4.    What was the original moisture content of the feed, before pre-drying?

*Response: You can find this information in Table 2.*

 5.    “Biocrude with high char contents could be considered a product from hydrothermal carbonization” (p. 6). This definition is quite weak and I suggest removing or rephrasing, as there are no fixed boundaries between different processes and, especially, the utilization of the products depends on the solutions utilized on the production plant and not merely by their amounts. For example, scaling up the process to a continuous flow unit could feature some form of on-line filtration allowing to recover important amounts of biocrude.

*Response: OK you are right, the phrase was removed.*

 6.    The section “Product analyses” tends to be redundant with respect to the section “Product recovery batch experiments”. For example, Karl-Fischer titration is described again. Moreover, azeotropic distillation is not defined. There seem to be two almost similar TOC apparatuses: please have it checked.

*Response: Yes, the text was a bit overly complicated. The section « Product recovery batch experiments »  was renamed to « Product recovery» to describe product recovery only. I have effectively simplified the text to limit descriptions of the actual equipment and procedures in the « Product analysis » section. The two TOC you refer to, are two different elements of the same system, one for liquids the other for solids.*

 7.    “A numerical value is attributed to the extraction method (…)”: this is not very clear, especially it is not clear how this value affects the regression algorithm.

*Response : This is just a modelling trick, as long as we have a numerical value we can use it in any regression correlation. Of course this works when there are two, a third would be complicated  as there we need  to be a physical sense to the variation in the value. An explanation was added to the text.*

 8.    The section “Data analysis with machine learning algorithms” seems quite didactical, even with some funny anecdote, like the story of the turkey. I would advise more conciseness, reporting only information necessary to an average-skilled reader to understand and reproduce the methodology.

*Response : After re-reading this section, we do believe that most of the text should stay. The concepts are not easy, and some theoretical background avoids most readers of having to dive into the references.*

 9.    In Fig. 5 caption, it should be specified that carbohydrates include lignin.

*Response: Done.*

 10.    The first part of the section “Continuous and batch experiments” belongs to Materials and Methods and not to Results.

*Response: Ok, as the conditions are globally the same for all experiments, some of it can be (and has been) moved to the Materials and Methods section.*

 11.    Units in Table 3 are wrong. For example, oil to char ratio is not a percentage, while char and gas yields in the last row are. The unit for yields should be made explicit (wt. %). A carbon balance to verify closure is highly advised.

*Response: Changes made. The carbon balance is presented in the text for the continuous experiment. Measurements were not made for batch experiments.*

 12.    One of the differences between batch and continuous is the long-lasting thermal transient in batch operations, which is not present in continuous.

*Response: Yes, this is mentioned in the text, in the section HTL Experiments. The text is moved towards the end, to make it more obvious.*

 13.    Fig. 6 is clearly wrong, as it is identical to Fig. 4. It should report the GC-MS analysis, instead.

*Response: Yes, this error was introduced between the proof approval and publication, the editor was notified. It is correct on the web version. Very strange.*

 14.    The caption in Fig. 7 is insufficient. Please explain what the left (a) and right (b) plots represent.

*Response: This section was removed from the manuscript.*

 15.    Eq. 3 and 4 look misleading. There should be a precise indication of the validity limit, especially for residence time. Moreover, it is awkward that a dependence is stated for residence time, when only two values of this variable were tested.

*Response: This section was meant to introduce the modelling part. It is to show how we can easily perform a linear regression with its limitations. You are of course right in that no firm conclusions can be drawn from this.*

 16.    At page 13, the paragraph: “From table 3 one could conclude (…) makes is even clear” is not clear and needs to be rephrased.

*Response: OK, rephrased.*

 17.    The legend in Fig. 8 is unclear. Its caption should report (a) and (b) instead of “left” and “right” (same also in Fig. 12).

*Response: This change was implemented on all double figures.*

 18.    Fig. 9 and 10 could be improved by drawing the parity line. In Fig.9 caption correct “calclated". Somewhere in the manuscript, a full account of the sources utilized to produce the dataset should be given.

*Response: Parity lined have been added to the graphs 9 and 10, old numbering. In the graphs in Figure 11 they have not been added as these graphs are already cluttered in the centre. The references to the data used are given in the Data Collection section. The actual data is given in the Excel file in the Zenodo reference as explained in is presented in the Data availabity/ Undelying Data section.*

 19.    The plot in Fig. 11 is not clear. What do the axes mean? What are their units of measure?

*Response: The text was amended to better explain this plot.*

 20.    What is “SHAP”?

*Response: SHAP means “Shapely Additive exPlanations” as is explained in the last paragraph of the section “Data analysis with machine learning algorithms”. Some more explanations were added to the text.*

 21.    The discussion in section “Feature analysis” is too much condensed and would probably need more space to be properly expanded. I would suggest reducing the number of graphs and selecting the information to comment.

*Response: The section was reworked and simplified with some more detailed explanations. The statistical noise is quite high and ferm conclusions are difficult to obtain. This in itself is however also a result.*

 22.    The reported explanation on why low values of lipids coincide with high values of carbohydrates is nicely obvious: “low values for lipids coincide with high values of carbohydrates due to the fact that high values of lipids correspond to low values of carbohydrates”. A more convincing explanation should be found, if any.

*Response: Yes as we mentioned in the previous response, this part has been reworked.*

 23.    Fig. 15 and 16 have insufficient captions, as each subplot must be identified and described. Moreover, the units of the represented variables must be given.

*Response: Figures have been removed after reconsidering this section.*

 24.    In the conclusions, “recycling of the HTL aqueous process water seems a good way to spare water resource”. This is misleading, as in a real process you would receive wet biomass, so no external water should be needed. On the contrary, there is a problem with the disposal of process wastewater according to environmental standards.

*Response: We have removed this section as it is not really important for the message we want to convey.*
